# Artificial Intelligence in Veterinary Education: Preparing the Workforce for Clinical Applications in Diagnostics and Animal Health

**DOI:** 10.3390/vetsci13020181

**Published:** 2026-02-12

**Authors:** Esteban Pérez-García, Ana S. Ramírez, Miguel Ángel Quintana-Suárez, Magnolia M. Conde-Felipe, Conrado Carrascosa, Inmaculada Morales, Juan Alberto Corbera, Esther SanJuan, Jose Raduan Jaber

**Affiliations:** 1 Grupo de Innovación Educativa VETFUN, Universidad de Las Palmas de Gran Canaria, 35001 Las Palmas de Gran Canaria, Spain; esteban.perezgarcia@ulpgc.es (E.P.-G.); anasofia.ramirez@ulpgc.es (A.S.R.); mmconde@uco.es (M.M.C.-F.); conrado.carrascosa@ulpgc.es (C.C.); esther.sanjuan@ulpgc.es (E.S.); 2 Departamento de Patología Animal, Producción Animal, Bromatología y Tecnología de los Alimentos, Universidad de Las Palmas de Gran Canaria, 35001 Las Palmas de Gran Canaria, Spain; inmaculada.morales@ulpgc.es (I.M.); juan.corbera@ulpgc.es (J.A.C.); 3 Departamento de Ingeniería Telemática, Universidad de Las Palmas de Gran Canaria, 35001 Las Palmas de Gran Canaria, Spain; 4 Departamento de Sanidad Animal, Universidad de Córdoba, 14071 Córdoba, Spain; 5 Departamento de Morfología, Universidad de Las Palmas de Gran Canaria, 35001 Las Palmas de Gran Canaria, Spain

**Keywords:** artificial intelligence, higher education, veterinary medicine, multimodal learning, educational innovation

## Abstract

Artificial intelligence (AI), including machine learning (ML) and deep learning (DL), is rapidly transforming clinical veterinary practice by enhancing diagnostics, disease surveillance and decision support processes across animal health domains. The safe and effective clinical deployment of these technologies, however, depends critically on the preparedness of the veterinary workforce, positioning veterinary education as a strategic enabler of translational adoption. This narrative review examines the integration of AI within veterinary education as a foundational step toward its responsible application in clinical practice. We synthesize current evidence on AI-driven tools relevant to veterinary curricula, including generative and multimodal large language models, intelligent tutoring systems, virtual and augmented reality platforms and AI-based decision support tools applied to imaging, epidemiology, parasitology, food safety and animal health. Particular attention is given to how the structured educational use of AI mirrors real-world clinical workflows and supports the development of competencies essential for clinical translation, such as data interpretation, uncertainty management, ethical reasoning and professional accountability. The review further addresses ethical, regulatory and cognitive considerations associated with AI adoption, including algorithmic bias, data privacy, equity of access and the risks of overreliance, emphasizing their direct implications for diagnostic reliability and animal welfare. By framing veterinary education as a controlled and reflective environment for AI engagement, this article highlights how pedagogically grounded training can facilitate safer clinical deployment, foster interdisciplinary collaboration and align technological innovation with professional standards in veterinary medicine.

## Simple Summary

Artificial intelligence (AI) is increasingly used in veterinary medicine to support diagnosis, disease surveillance and clinical decision-making. To ensure that these technologies are applied safely and responsibly in practice, veterinarians must be adequately trained during their education. This review explores how AI is currently being incorporated into veterinary curricula and how the educational use of AI can prepare students for future clinical applications. We describe the main types of AI tools used in veterinary education, including generative and multimodal models, virtual and augmented reality, and decision support systems across areas such as imaging, epidemiology, parasitology, food safety and animal health. The review also highlights important challenges related to ethics, data privacy, bias and overreliance on AI. Overall, we show that veterinary education plays a key role in bridging technological innovation and clinical practice, helping future veterinarians to develop the skills needed to use AI in a safe, ethical and clinically meaningful way.

## 1. Introduction

Artificial intelligence (AI) has rapidly evolved into a transformative technological force capable of performing tasks traditionally associated with human cognitive functions, including learning, reasoning, problem solving and language comprehension [1]. Its foundation rests on a broad spectrum of computational approaches—machine learning, deep learning, natural language processing and robotics—that enable computer systems to analyze information, extract patterns and adapt responsively to new data [2,3]. These capabilities have contributed to reshaping sectors as diverse as healthcare, finance, manufacturing, transport and entertainment, with education emerging as one of the fields experiencing the most profound and rapid shifts [2,3,4].

In public perception, the term “AI” is often narrowly associated with high-profile tools such as ChatGPT, which are frequently the only systems known to non-specialist users [5,6]. However, the current ecosystem of generative AI is considerably broader, comprising multiple leading providers (OpenAI, Google, Anthropic, Microsoft, DeepSeek and Meta), each contributing models with distinctive multimodal, analytical or integrative capabilities suited for educational contexts [7,8,9].

Recent developments in the field highlight the increasing importance of multimodal AI, with the ability to process text, images, audio and video within a unified analytical framework. For veterinary education, which relies heavily on the interpretation of clinical images, anatomical structures, laboratory data and animal behavior, multimodality represents a highly valuable pedagogical asset [10]. It allows the construction of realistic scenarios that enrich students’ learning experiences and bring them closer to the complexity of professional practice.

The integration of AI into university teaching has expanded significantly, with applications that support personalized learning, intelligent tutoring, automated evaluation, simulation-based training, clinical reasoning support and efficient academic management [11]. In disciplines such as anatomy, pathology, animal health and food technology (core areas of the veterinary curriculum), AI has the potential to augment traditional pedagogical approaches by providing students with dynamic models, interactive case studies and real-time feedback that enhance their conceptual understanding and practical reasoning.

The rapid growth of AI in veterinary education demands not only an exploration of its pedagogical benefits but also critical reflection on its risks and limitations. Ethical concerns (including data privacy, algorithmic bias, equity of access, academic integrity and the potential erosion of students’ independent reasoning) must be addressed to ensure that AI supports rather than replaces human expertise in clinical decision making [12]. Furthermore, regulatory frameworks in animal health and veterinary medical practice add a layer of complexity that institutions must navigate with care.

This article is conceived as a focused narrative review rather than an empirical educational research study. Its objective is not to test a specific pedagogical intervention or formulate a hypothesis-driven research question but to synthesize current evidence, conceptual frameworks and emerging applications of artificial intelligence relevant to veterinary education and their implications for future clinical practice.

Accordingly, this review is guided by the overarching question of how veterinary education can equip future graduates with the competencies required to use artificial intelligence responsibly, critically and effectively in clinical veterinary practice.

Against this backdrop, this narrative review aims to

1. Analyze the impact of AI on university-level pedagogy, with an emphasis on personalized and autonomous learning;
2. Explore the multimodal applications of contemporary AI models across core areas of veterinary education, including animal health, anatomy, parasitology and food science;
3. Outline the ethical, pedagogical and regulatory considerations necessary for the responsible integration of AI into veterinary curricula.

In this context, veterinary education emerges as a critical bridge between rapid technological innovation and the safe, effective deployment of artificial intelligence in clinical veterinary practice. As AI, machine learning and deep learning systems become increasingly embedded in diagnostics, epidemiological surveillance, disease management and decision support workflows, the competence of future veterinarians to critically interpret, validate and appropriately apply these tools becomes essential [13]. Educational exposure to AI-based technologies not only familiarizes students with their technical capabilities but also shapes their clinical reasoning, ethical awareness and professional judgment. Framing veterinary education as a foundational enabler of clinical AI adoption, this review aims to synthesize current evidence on AI applications relevant to veterinary training and to discuss how the pedagogically grounded integration of AI can directly support reliable diagnostics, animal health management and responsible clinical decision making.

## 2. Materials and Methods

This study was conducted as a narrative review with scoping elements, explicitly designed to synthesize and contextualize the existing literature, rather than to conduct empirical educational research [14]. Accordingly, this review does not follow a protocol-driven systematic or PRISMA-based scoping methodology but adopts a flexible and interpretative approach appropriate for synthesizing a rapidly evolving and heterogeneous body of literature. A comprehensive literature search was carried out using Scopus, Web of Science, ERIC, PubMed and Google Scholar, focusing primarily on publications from 2020 to 2025 in order to capture the most recent developments in AI-supported educational practices. Search terms included combinations of “artificial intelligence”, “generative AI”, “ChatGPT”, “large language models”, “autonomous learning”, “veterinary education”, “health professions education”, “higher education”, “simulation” and “multimodal learning”.

Peer-reviewed journal articles, narrative and systematic reviews and selected conference papers written in English were considered eligible when they addressed the use of AI tools, platforms or pedagogical frameworks in higher education, with particular relevance to veterinary and health sciences curricula. Non-peer-reviewed sources, such as institutional reports, policy documents and industry documentation, were selectively included to contextualize emerging technologies when peer-reviewed evidence was limited. All retrieved records were imported into the Mendeley reference management software version 2.139.0 (Elsevier) to organize the dataset, manage references and remove duplicate records. Articles were screened for relevance based on titles, abstracts and full-text evaluation and subsequently analyzed thematically to identify key educational applications, pedagogical implications, ethical considerations and future research directions. This approach was adopted to provide a broad yet structured overview of a rapidly evolving field.

The literature synthesis followed a structured and transparent process. Source selection and thematic inclusion were guided by relevance to the review objectives rather than by predefined exclusion criteria, allowing the integration of educational, technological and domain-specific perspectives relevant to veterinary training. Thus, a clear conceptual scope was defined in alignment with three overarching objectives: (1) to examine the impact of AI on personalized learning in higher education; (2) to synthesize concrete multimodal applications of AI within core areas of the veterinary curriculum—specifically animal health, anatomy, parasitology and food science; and (3) to develop a framework addressing the ethical, pedagogical and regulatory considerations necessary for the responsible implementation of AI in veterinary teaching. This scope enabled the integration of both educational research and domain-specific applications within the biomedical and veterinary sciences.

A non-systematic but comprehensive search strategy was then applied across scientific literature databases and specialized sources, with a particular emphasis on publications from 2024–2025, reflecting the accelerated pace of innovation in generative and multimodal AI. The review incorporated peer-reviewed journal articles, narrative and systematic reviews, conference proceedings, white papers and policy documents from recognized AI developers, as well as institutional and governmental guidelines relevant to veterinary and medical education. Although the search was not protocol-driven in the manner of a systematic review, particular care was taken to ensure both breadth and recency, especially regarding the capabilities, limitations and pedagogical implications of emerging AI models.

Extracted information was subsequently organized into major thematic domains, including (i) educational transformations associated with AI adoption (such as personalization, intelligent tutoring, assessment and learning analytics); (ii) technological developments in generative and multimodal AI (including GPT-4/4o, Gemini 2.5, Claude 4, Copilot, DeepSeek V3/R1 and Llama 4); (iii) discipline-specific applications within veterinary education; and (iv) ethical, regulatory and professional considerations. Representative examples and illustrative case studies, particularly those highlighting multimodal functionality or real-world integration within veterinary programs, were selected to support each thematic area.

Finally, thematic synthesis was conducted to connect disparate strands of the literature and generate an integrated, cross-disciplinary understanding of the role of AI in veterinary education. This synthesis highlights recurring trends, emerging benefits and persistent limitations, while highlighting the alignment between technological advances and the competencies required for contemporary veterinary practice. Overall, the methodological approach adopted ensures that this review captures both the breadth and depth of current developments, providing an up-to-date, coherent and analytically rigorous perspective on AI as a support tool for autonomous learning in veterinary education.

## 3. AI in Higher Education: Expanding Context and Pedagogical Paradigms

Artificial intelligence has begun reshaping higher education at a rapid pace, altering not only the delivery of content but also the underlying pedagogical frameworks that guide teaching and learning. Universities worldwide are increasingly adopting AI to enhance instructional quality, personalize learning experiences and streamline administrative processes [15]. Veterinary education, with its multidimensional integration of biological, clinical and technological knowledge, appears particularly well positioned to benefit from these developments.

Recent years have been described as a critical inflection period for AI adoption in education. What once seemed a distant vision of intelligent classrooms and automated pedagogical systems has rapidly materialized into practices with effects across diverse contexts. Available reports and surveys suggest that a large proportion of educational institutions have integrated AI into at least one area of instruction, reflecting a systemic shift toward digitalized and data-informed learning environments [16]. Beyond its role as a supportive technology, AI is increasingly described as a pedagogical partner capable of influencing curriculum design, learning strategies and assessment methodologies. Large-scale analyses of instructor–AI interactions show that a substantial proportion of educators now rely on AI for curriculum development and academic tasks, underscoring the depth of its integration into higher education practice [17]. Although large-scale studies provide quantitative indicators on the adoption of AI technologies in higher education and health-related disciplines, quantitative data specifically focused on veterinary and animal sciences remain limited. In most cases, veterinary education is embedded within broader biomedical or health sciences datasets, highlighting a current evidence gap regarding discipline-specific adoption rates and usage patterns [18].

One of the most frequently discussed pedagogical implications of AI lies in its ability to create personalized learning environments. AI-driven systems are reported to analyze student performance patterns, learning styles and behavioral indicators to dynamically adjust instructional content. This adaptability may be particularly valuable in veterinary education, where students often enter programs with heterogeneous backgrounds in anatomy, diagnostics and laboratory skills. Adaptive learning platforms and intelligent tutoring systems have been associated with individualized feedback, differentiated instructional pathways and enhanced learner engagement in reported studies [19,20,21,22]. By adjusting the difficulty, pacing and modality, such systems may approximate forms of personalized instruction traditionally associated with one-to-one mentorship.

In parallel, intelligent tutoring systems and AI-supported assessment tools have likewise gained prominence in higher education discourse. Through natural language interaction, contextual questioning and iterative feedback, these systems are designed to simulate aspects of human tutoring, particularly in large cohorts where individualized attention is limited [19]. Automated evaluation tools are increasingly used to support grading, identify learning gaps and assist in standardizing assessment practices across diverse educational settings [20,21]. In veterinary education, their relevance is often discussed in relation to courses that require timely feedback to support the development of clinical and diagnostic reasoning, although their pedagogical impact remains context-dependent.

Beyond direct instructional functions, AI is also reported to contribute to administrative efficiency and academic support within higher education institutions. Intelligent assistants and chatbots may support admissions processes, course registration, timetable optimization and student services, potentially reducing operational burdens on faculty and enabling greater focus on teaching and student engagement [23]. AI-driven learning analytics platforms are increasingly used to monitor student progress at scale, identify patterns of disengagement and inform early interventions, although their effectiveness depends on institutional implementation and governance.

The growing presence of AI in educational contexts has also prompted renewed attention to AI literacy and critical digital competencies. Understanding how AI systems function, and their limitations, ethical implications and societal impact, is increasingly recognized as an essential component of higher education [24,25]. Within veterinary programs, the literature emphasizes the importance of developing not only technical familiarity with AI tools but also the capacity to critically evaluate AI-generated outputs and recognize situations in which professional judgment must override algorithmic recommendations. AI-assisted learning has been discussed within constructivist educational frameworks, where AI functions as a cognitive scaffold supporting learners as they progress toward greater autonomy. While some studies suggest that large language models may enhance motivation and comprehension under appropriate supervision [26], concerns persist regarding their potential influence on clinical reasoning and the risk of overreliance [27].

To counterbalance the potential dependency on AI systems, pedagogical strategies that promote deep learning and critical thinking are widely considered important. These include creative assignments emphasizing synthesis over reproduction, narrative feedback approaches, concept mapping and hands-on simulations that foster transferable skills. Such strategies align with calls for adaptive mastery in AI-enabled educational ecosystems, emphasizing flexible and critically informed professional competence rather than mere content acquisition [28]. Established pedagogical frameworks further support the structured integration of AI in veterinary education. The Technological Pedagogical Content Knowledge model highlights the intersection of technological tools, pedagogical strategies and domain-specific veterinary content [29,30], while the Substitution, Augmentation, Modification, Redefinition framework conceptualizes AI applications along a continuum from substitution to pedagogical redefinition [31]. Cognitive Load Theory informs the design of AI-enhanced learning environments, particularly in immersive VR/AR contexts [32,33], and Multimodal Learning Theory underscores AI’s capacity to integrate text, images, audio and video in ways that may enhance retention and comprehension [34,35].

Across the veterinary curriculum, AI-enhanced educational applications have been described as increasingly resembling the data-intensive, multimodal and decision-oriented nature of contemporary clinical practice [36]. Exposure to AI-supported workflows in epidemiology, imaging, parasitology, food safety and animal health may help students to develop practical skills relevant to diagnostics, disease management and public health interventions. By integrating these tools within structured educational contexts, veterinary programs can facilitate the safe translation of AI innovations from the classroom to the clinic, supporting reliable decision making and safeguarding animal welfare.

**Figure 1:**
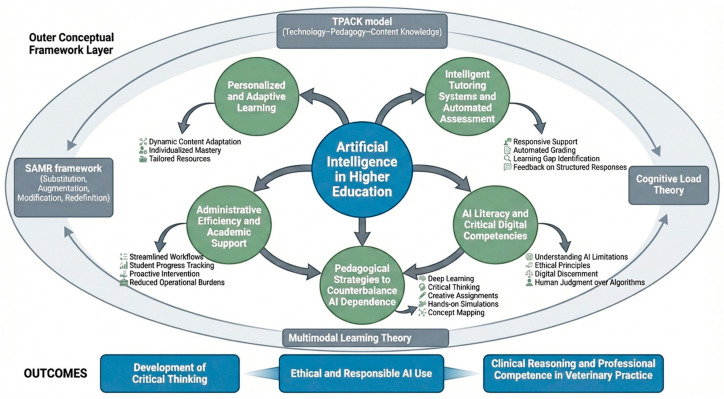
Conceptual framework of artificial intelligence integration in higher education and veterinary pedagogy.

To synthesize the multiple dimensions discussed above, Figure 1 presents a conceptual framework illustrating the integration of artificial intelligence within higher education, with a specific emphasis on veterinary education.

## 4. Overview of Major Generative AI Models

### 4.1. OpenAI (GPT-4, GPT-4o)

OpenAI is widely described as a leader in the field of generative AI, with GPT-4 and GPT-4o being among the models most frequently discussed in educational contexts. These models are notable for their native multimodal capabilities, allowing them to process radiographs, analyze laboratory results, evaluate clinical images and generate domain-specific explanations relevant to veterinary sciences [7].

GPT-4o has been reported to further enhance real-time reasoning, enabling the integration of text, image and audio inputs. Such capabilities may support the construction of comprehensive clinical cases, anatomical visualizations and practical scenarios commonly used in veterinary teaching.

### 4.2. Google (Gemini 2.5)

Google’s Gemini 2.5 is often described as a highly advanced multimodal model, particularly in relation to the processing of technical documentation and the analysis of medical and scientific imagery. Its reported strengths include detailed document analysis and image interpretation, which may be relevant in educational settings requiring structured information synthesis. One pedagogical advantage frequently noted in the literature is its integration with Google Workspace, facilitating deployment within institutions already relying on Google-based digital infrastructures. Gemini’s capabilities in document extraction and summarization have been discussed as supporting the development of structured learning materials and evidence-informed educational activities [37].

### 4.3. Anthropic (Claude 4)

Claude 4 is characterized in the literature by its emphasis on safety, factual accuracy and transparent reasoning—features considered particularly important in health-related educational contexts. Its capacity to process extended textual inputs (up to 200,000 tokens) allows for large-context analysis, which may make it suitable for working with textbooks, large case files and multi-section academic documents [38,39].

Its systematic reasoning style has been discussed as potentially useful in co-tutoring scenarios that involve a sustained technical dialog, with the aim of supporting higher-order thinking while reducing the risk of hallucinated outputs.

### 4.4. Microsoft (Copilot and Copilot Chat)

Microsoft has integrated generative AI capabilities into the Office 365 ecosystem, offering a practical option for educational institutions already using platforms such as Word, PowerPoint, Teams or OneNote. Within this environment, Copilot has been described as supporting the automated summarization of academic documents, the generation of didactic materials, the analysis of student assignments and course planning activities [40]. Its widespread availability and integration into existing university workflows are frequently cited as factors that may facilitate large-scale adoption in higher education settings.

### 4.5. DeepSeek (V3, R1)

DeepSeek has emerged as an alternative to established commercial models, with reports highlighting its focus on computational efficiency and reduced operational costs [41]. DeepSeek V3 is described as being oriented toward technical analysis, while DeepSeek R1 has been associated with enhanced reasoning capabilities. These features may be of interest to educational institutions operating under resource constraints.

### 4.6. Meta (Llama 4)

Llama 4 is notable for its exceptionally large context window, reported to reach up to 10 million tokens, allowing the processing of extensive textual material such as entire books, large datasets or long-running dialogs. As an open-source model, Llama enables educational institutions to develop customized AI systems tailored to specific species, curricular requirements or clinical domains [42]. This flexibility is frequently discussed as advantageous in contexts requiring campus-controlled data governance, enhanced privacy protections or domain-specific terminology.

#### Contextual Synthesis

To contextualize the role of emerging AI systems in veterinary education, Table 1 summarizes the core strengths, pedagogical applications and distinctive features of the leading generative AI models available in 2025.

Understanding the strengths, limitations and contextual suitability of generative AI models is important for their translation into veterinary education and, ultimately, clinical practice. The literature suggests that these systems offer considerable potential for processing large and heterogeneous data streams; integrating textual, visual and structured clinical information; and supporting tasks such as diagnostic imaging interpretation, clinical documentation, epidemiological trend analysis and decision support activities. At the same time, their outputs remain highly dependent on data quality, prompt formulation and contextual alignment and may be affected by limitations related to bias propagation, reduced transparency and the absence of true clinical reasoning or causal understanding. Educational engagement with generative AI may therefore serve not only to familiarize future veterinarians with emerging tools but also to develop critical evaluation skills, enabling the responsible, accountable and clinically reliable use of AI technologies within well-defined professional boundaries.

## 5. Specialized Educational Agents and Platforms

A parallel development alongside generative models is the rise of platforms specifically designed for pedagogical purposes. These tools do not merely repurpose general LLM capabilities—they embed instructional design principles, citation grounding or real-time feedback mechanisms that directly support teaching and learning.

Recent evidence indicates that, among specialized educational AI platforms, NotebookLM is currently the only system with clearly documented real-world implementations in higher education. In health professions education, NotebookLM has been used as a source-grounded instructional support tool, allowing faculty to upload guidelines, lecture notes and multimodal materials to generate teaching resources, refine curricular content and support collaborative content development among instructors and students. Educators report improved efficiency and pedagogical flexibility, although formal learning-outcome assessments remain limited [43].

In a separate implementation within physics teacher education, NotebookLM was deployed as a Socratic, retrieval-augmented tutoring system, where instructors curated authoritative source documents and constrained student interaction to citation-grounded dialog. This design supported guided problem solving and traceable explanations while reducing ungrounded content generation, highlighting the platform’s potential for structured, instructor-controlled learning support [44].

### 5.1. Google NotebookLM

NotebookLM has rapidly become one of the most innovative tools for educational use, leveraging Gemini’s multimodal architecture to act as a document-grounded learning assistant [37,45].

Its defining feature, source grounding, ensures that all responses are strictly based on instructor-selected materials, making it exceptionally useful for the following:

- Summarizing veterinary pathology notes;
- Synthesizing epidemiology protocols;
- Comparing anatomical structures;
- Guiding students through curated reading lists.

This approach may help to reduce hallucinations and enhance academic transparency.

### 5.2. ChatGPT Study Mode

Introduced in 2025, Study Mode transforms ChatGPT into a Socratic personal tutor [46]. Rather than giving direct answers, the system supports deep learning by posing guiding questions, encouraging reflection and stimulating metacognitive skills [47].

Its functions include

- Breaking down complex scientific concepts into step-by-step reasoning;
- Generating practice questions;
- Adapting interactions to each learner’s history;
- Supporting difficult subjects such as physiology, parasitology or microbiology.

Study Mode’s inquiry-based design aligns with veterinary education’s need for applied reasoning and case-based learning.

### 5.3. Microsoft 365 Copilot Agents

Copilot Chat includes customizable agents designed for institutional deployment. These agents can embody specific roles (such as instructional designer, lab assistant or assessment specialist) and support academic workflows by generating learning materials, analyzing student performance or offering targeted feedback [40].

This modular architecture allows veterinary faculties to integrate curated AI agents into teaching laboratories, anatomy sessions or epidemiology courses.

### 5.4. Claude for Education

Anthropic’s educational version of Claude incorporates a dedicated Learning Mode emphasizing reflective thinking and conceptual reasoning [38,39].

Instead of straightforward explanations, Claude often answers with probing questions designed to deepen understanding. This aligns with the philosophical foundations of veterinary case-based teaching, where diagnostic reasoning must be meticulously justified.

### 5.5. Meta AI (Llama 4 Scout and Maverick)

The Llama 4 suite includes models trained under a Mixture of Experts (MoE) architecture, enhancing efficiency in processing text, images and videos [42].

Educational applications have been demonstrated by Blended Labs [48], which uses Llama-based AI to

- Automatically grade handwritten assignments;
- Support language and STEM instruction;
- Provide 24/7 co-teaching assistance;
- Deliver personalized feedback correlated with measurable performance improvements.

These capabilities are highly relevant for veterinary anatomy, clinical skills training and laboratory-intensive courses.

### 5.6. Perplexity AI

Perplexity has established itself as a conversational academic search engine, combining deep research features with real-time citation mechanisms [49].

Educators value its

- Transparent citation system;
- Up-to-date academic source access;
- Ability to alternate between specialized models;
- Suitability for undergraduate students from age 13 onward.

It fosters critical research skills that are essential for veterinary students engaging in evidence-based practice.

Specialized educational AI agents and platforms represent an intermediate step between general-purpose models and real-world clinical applications. By embedding a pedagogical structure, source grounding and reflective interaction, these systems mirror key features of clinical decision support tools used in veterinary practice. Training students to interact with such platforms fosters competencies that are directly transferable to clinical environments, including evidence-based reasoning, transparency in information sourcing and the critical evaluation of AI-assisted recommendations [50].

## 6. Applications of Artificial Intelligence Across the Veterinary Curriculum

Artificial intelligence has been increasingly discussed in the literature as a versatile support tool across multiple areas of the veterinary curriculum. Given that veterinary medicine integrates biological sciences, clinical reasoning, public health, pathology, parasitology and food safety, the inclusion of AI has been proposed as a means of supporting complex, multimodal and data-intensive learning activities that closely reflect real-world professional practice. Accordingly, this section provides a comprehensive overview of AI applications across major subject areas, highlighting their pedagogical value, technological underpinnings and relevance for future veterinary graduates [51].

**Figure 2:**
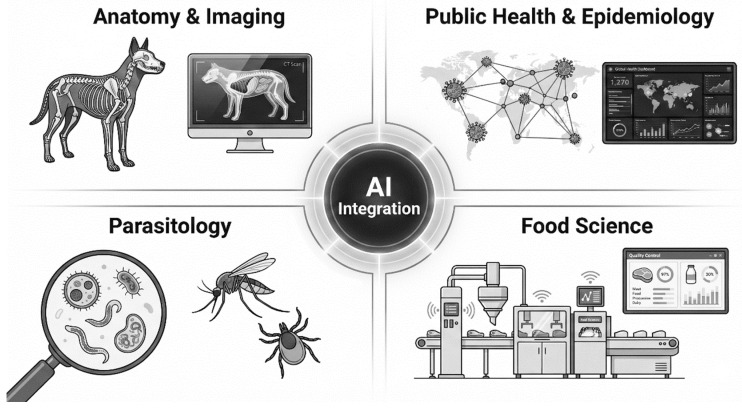
Schematic overview of AI integration across core veterinary disciplines, including anatomy and imaging, parasitology, food science and public health.

AI applications are described across multiple domains within the veterinary curriculum, including anatomy, imaging, parasitology, food science and public health. Figure 2 offers an overview of these interconnected areas and their relationships within AI-supported veterinary education.

### 6.1. Veterinary Public Health and Animal Health

Animal health is a foundational area of veterinary education, encompassing disease prevention, diagnosis, epidemiology, surveillance and public health. The integration of AI into this domain has been discussed in the literature as a potential means of supporting both theoretical understanding and practical skill development, through predictive modeling, simulation-based learning and intelligent tutoring approaches.

#### 6.1.1. Epidemiology and Digital Surveillance

AI-driven approaches have been described as offering sophisticated tools for exploring disease dynamics, prediction and mitigation strategies. Machine learning algorithms can be used to analyze complex datasets that include environmental, genomic and spatial information, enabling students to explore realistic surveillance workflows in educational settings [52]. Through these systems, learners may be exposed to the intricacies of outbreak dynamics, early detection methods and evidence-based decision making.

Predictive models are frequently employed in educational simulations; this allows hands-on practice with simulated outbreaks, including scenarios involving porcine influenza or salmonellosis, where student teams may be tasked with implementing sampling strategies and designing intervention plans and receive automated feedback based on algorithmic simulations [53]. These approaches are often discussed in relation to the emerging concept of precision veterinary epidemiology, which emphasizes the integration of multiple digital data sources to support timely and cost-effective disease control.

#### 6.1.2. Infectious Diseases and Diagnostic Support

AI-based diagnostic support systems have been proposed as educational tools for illustrating concepts such as test sensitivity, specificity and decision thresholds. Integrating these tools into veterinary education may improve the teaching of diagnostic reasoning by enabling students to examine how algorithmic outputs align—or conflict—with classical epidemiological inferences [36].

AI-generated cases are increasingly reported to simulate clinical interviews, infectious disease scenarios and zoonotic transmission pathways [54]. These simulation environments can provide immediate feedback and may help students to explore the complexities of host–pathogen interactions, clinical symptomatology and intervention strategies within a controlled educational framework.

#### 6.1.3. Preventive Medicine and Risk Stratification

Preventive veterinary medicine has been identified as an area that may benefit from AI-supported strategies that allow students to model risks, predict outcomes and evaluate the impacts of different interventions. Decision tree algorithms are often used pedagogically to guide learners through scenarios involving sampling strategies, vaccination prioritization or biosecurity protocols, illustrating how recommendations shift based on data quality or classification thresholds [55]. These types of exercises are described as fostering critical thinking by highlighting the consequences of data limitations and uncertainty—concepts that are central to responsible clinical and public health decision making.

#### 6.1.4. Veterinary Public Health and Emergency Readiness

AI tools have been described as supporting scenario-based learning in the context of public health crises, including outbreak containment, surveillance integration and the evaluation of non-pharmaceutical interventions [56]. Virtual dashboards, predictive models and interactive simulations are commonly used to explore preparedness strategies and may help to illustrate how rapidly evolving data can inform public health decision making.

Taken together, these applications suggest that AI may contribute to veterinary public health education by exposing students to complex, authentic problem-solving environments within a controlled educational setting.

### 6.2. Anatomy and Medical Imaging

Anatomy is central to the formation of veterinary professionals, requiring a detailed understanding of structural relationships and the ability to interpret clinical images. AI-driven tools are increasingly discussed as supporting both foundational instruction and more advanced diagnostic training within anatomy-focused curricula.

#### 6.2.1. Image Interpretation and Predictive Modeling

Machine learning and deep learning methods, particularly convolutional neural networks (CNNs), have been widely applied to the automated interpretation of radiographic, CT and MRI images. In educational and clinical contexts, these models may assist in identifying fractures, tumors and structural abnormalities, thereby supporting the development of diagnostic reasoning while expanding their exposure to diverse clinical cases [18].

In orthopedic applications, CNNs have been used to predict hip and elbow dysplasia in young dogs based on pelvic radiographs. Such applications may help learners to visualize relationships between anatomical features and clinical outcomes, and they can support the early recognition of pathological changes within training scenarios [57].

#### 6.2.2. Surgical Planning, 3D Models and Robotics

AI-enhanced imaging has also been discussed as supporting surgical planning and personalized prosthesis design. When combined with 3D reconstruction and modeling, AI allows learners to examine individualized anatomical variations and consider how structural differences influence surgical approaches. Advances in medical robotics further illustrate the convergence of AI with interventional practice. Within educational contexts, these developments are often presented as offering students a forward-looking perspective on emerging clinical technologies and innovation pathways [58].

#### 6.2.3. Interactive and Immersive Anatomical Education

Virtual reality and augmented reality environments powered by AI have been described as supporting the exploration of dynamic 3D anatomical models. These platforms allow students to engage with anatomical structures, visualize spatial relationships and simulate dissection-like experiences within a risk-free educational environment [8,24].

Large language models such as ChatGPT are increasingly discussed as functioning as virtual tutors, offering case-based reasoning and detailed explanations tailored to students’ questions. Within educational contexts, these tools may support autonomous learning, particularly when addressing anatomically complex regions such as the head, neck and thorax. Collectively, these AI-enhanced have been proposed as contributing to the modernization of anatomical education by combining interactive technologies with guided reasoning approaches.

### 6.3. Parasitology and Parasitic Diseases

Parasitology education aims to prepare future veterinarians to prevent, diagnose and manage parasitic infections. Within this domain, AI has been introduced as a set of tools that may support microscopic identification, lifecycle comprehension and scenario-based problem-solving activities.

#### 6.3.1. Automated Identification and Prediction

AI models have been reported to show promising performance in identifying parasites and vectors, such as mosquito species or Echinococcus multilocularis lesions, supporting diagnostic accuracy [59,60]. Predictive algorithms have also been applied to tasks such as modeling parasite protein structures or forecasting infection risks [2,3]. In educational settings, these tools may allow students to practice the recognition of morphological features and biological markers across a wide range of parasitic species.

#### 6.3.2. Simulation-Based Learning and Virtual Reality Parasites

Immersive VR platforms have been described as enabling students to explore parasitic lifecycles, host interactions and infection mechanisms within interactive learning environments. Some studies report associations with improved knowledge retention and increased student engagement when VR-based approaches are compared with traditional lecture formats [61,62,63]. Illustrative examples include simulations of *Plasmodium falciparum* invasion or virtual escape room designs that challenge learners to infer immune response mechanisms under guided conditions [64].

#### 6.3.3. ChatGPT and AI-Based Assessment

AI chatbots have been explored as tools for generating multiple-choice questions, delivering explanations and supporting formative assessment in parasitology courses [65]. However, the literature also highlights limitations in highly specialized or advanced examinations, underscoring the need for supervised use and domain-specific calibration [66].

### 6.4. Food Science, Food Technology and Food Safety

Food science is an essential area in veterinary education, linking microbiology, processing technologies, hygiene, regulation and public health. AI has been discussed as offering a range of educational applications, including the simulation of industrial environments, process exploration and support for microbiological analysis.

#### 6.4.1. AI in Teaching and Learning Food Science

AI has been reported to support literature searching, project structuring and critical reflection among students. It may facilitate educational strategies such as data-driven learning, role-playing exercises and predictive modeling projects, which can help learners to engage with real-world food engineering challenges [67,68]. These approaches allow students to explore practical scenarios involving rheology, processing parameters and quality control within controlled learning settings.

#### 6.4.2. Food Hygiene and Virtual Laboratories

Virtual laboratories and AR-based environments have been described as enhancing the conceptual understanding of hygiene protocols while avoiding the risks inherent in real laboratory settings. Several studies report improvements in practical skill development and conceptual retention when VR/AR tools supported by AI are incorporated into teaching activities [69,70].

Microbiological predictive tools—such as ComBase and the Food Spoilage and Safety Predictor—are commonly used in educational contexts to allow students to explore microbial growth and inactivation kinetics by applying theoretical concepts to realistic datasets [71].

#### 6.4.3. AI in Food Processing and Quality Control

In food technology and engineering education, AI has been applied to support process optimization exercises, resource management scenarios and quality assessment activities. AI-driven predictive models may enable students to evaluate efficiency, design safer products and perform environmental impact assessments within simulated contexts [72]. Bibliometric analyses suggest that AI is increasingly influencing emerging trends in food engineering, highlighting growing intersections with machine learning, sensing technologies and automation [73]. Together, these applications illustrate how AI may contribute to linking scientific knowledge with industry-relevant competencies within veterinary food science education.

### 6.5. Multimodality as a Core Pedagogical Asset

**Figure 3:**
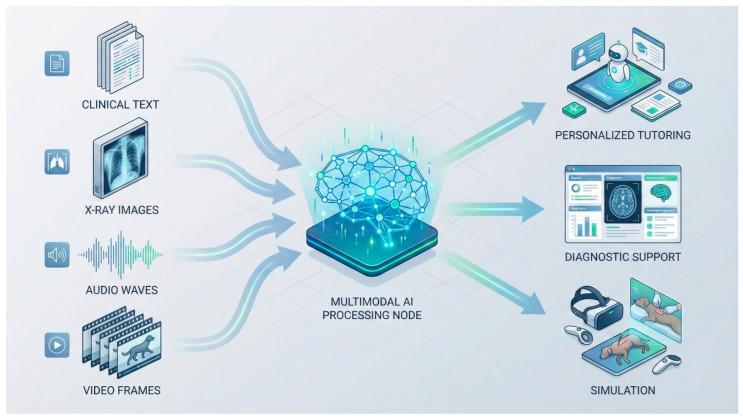
Representation of a multimodal AI processing architecture illustrating how clinical text, X-ray images, audio data and video frames can be integrated to generate educational outputs such as diagnostic support, personalized tutoring and simulation environments.

The integration of text, clinical imagery, audio signals and video data into unified multimodal processing frameworks is widely recognized as central to the pedagogical potential of contemporary AI systems (Figure 3). This capacity is discussed as enriching clinical reasoning processes by reflecting the complexity and data integration demands of real-world veterinary practice.

Across veterinary disciplines, multimodal AI is increasingly described as a core capability within educational and clinical contexts. Its ability to integrate text, images, audio and video has been highlighted as enabling the more comprehensive interpretation of complex datasets typical of veterinary practice.

Illustrative examples discussed in the literature include the following:

- Combining textual case descriptions with radiographs and bloodwork;
- Analyzing ultrasound clips alongside clinical signs;
- Evaluating heart sound recordings with ECG traces;
- Interpreting videos of animal movement to assess neurological function.

Taken together, these integrative approaches are described as supporting cohesive learning experiences that approximate authentic clinical scenarios, reinforcing diagnostic reasoning and practical judgment.

Across the veterinary curriculum, AI-enhanced educational applications are frequently reported as reflecting the data-intensive, multimodal and decision-oriented nature of contemporary clinical practice. In particular, artificial intelligence has been extensively applied and discussed in veterinary diagnostic imaging, where machine learning models analyze large and heterogeneous datasets derived from radiography, ultrasonography, computed tomography and magnetic resonance imaging to support clinical interpretation in educational and clinical research contexts [74].

Similarly, deep learning approaches have been adopted and explored across veterinary diagnostics and animal health to integrate imaging data with clinical records and laboratory information, thereby reflecting the multimodal complexity of real-world veterinary workflows, as described in recent reviews [75].

Beyond diagnostics, AI-supported systems have been increasingly examined in epidemiology, disease surveillance and animal health management, where large-scale data analysis may enable the early detection of disease patterns and support population-level and public health interventions [76].

In addition, applications of artificial intelligence in veterinary clinical pathology have been described as illustrating the decision-oriented nature of modern clinical practice by assisting in the interpretation of hematological, biochemical and cytological data, thereby supporting diagnostic reasoning and disease management [77].

### 6.6. From Educational Use to Clinical Deployment

The transition from educational applications of artificial intelligence to its safe and effective use in clinical veterinary practice is neither automatic nor trivial. While AI tools are becoming increasingly accessible in academic settings, their clinical deployment requires veterinarians who can critically assess algorithmic outputs, understand model limitations and integrate AI-assisted insights within established diagnostic and decision-making frameworks.

Educational exposure to AI-driven systems plays a central role in this translational process. Training students with AI-supported diagnostic simulations, multimodal case analyses and decision support exercises familiarizes them with workflows that closely resemble those encountered in clinical environments [78]. Such experiences foster competencies that are essential for clinical practice, including data interpretation, uncertainty management, ethical reasoning and accountability in decision making.

Importantly, the structured educational use of AI allows future veterinarians to develop an informed understanding of algorithmic biases, species-specific data limitations and context-dependent performance variability [79]. These aspects are particularly relevant in veterinary medicine, where interspecies differences, heterogeneous datasets and evolving epidemiological conditions can significantly influence model reliability. Early recognition of these constraints supports safer clinical adoption and helps to prevent inappropriate reliance on AI-generated recommendations.

By positioning veterinary education as a controlled and reflective environment for AI engagement, academic institutions can serve as gatekeepers for responsible clinical translation. Graduates trained under pedagogically grounded AI frameworks are better equipped to collaborate with computer scientists and bioengineers, contribute to the validation of clinical AI tools and ensure that technological innovation aligns with professional standards, regulatory requirements and animal welfare considerations [80].

Across the veterinary curriculum, AI-enhanced educational applications increasingly mirror the multimodal, data-intensive and decision-oriented nature of contemporary clinical practice. When integrated within structured pedagogical frameworks, these tools support the progressive development of competencies required for AI-assisted diagnostics, disease management and public health decision making. By aligning educational use with clinical realities, veterinary programs can facilitate the safer and more effective translation of AI innovations from the classroom to the clinic, reinforcing professional judgment, diagnostic reliability and animal welfare.

## 7. Ethical, Pedagogical and Clinical Considerations of AI Integration

As artificial intelligence becomes increasingly embedded in educational ecosystems, concerns surrounding ethics, equity, academic integrity and professional responsibility have been widely identified as central issues for veterinary institutions [81]. While AI offers substantial pedagogical potential, its integration is generally regarded as requiring careful consideration in order to avoid unintended consequences that may compromise learning quality, cognitive development or ethical standards. This section examines key challenges and principal risks associated with AI adoption in veterinary clinical education, drawing on international guidelines, empirical evidence and professional recommendations.

One of the most frequently discussed challenges relates to the cognitive impact of AI and the risk of overreliance on automated systems. Although AI can effectively support grading, feedback and instructional scaffolding, emerging evidence suggests a gap between tasks that can be automated and those that educators and clinicians consider pedagogically and clinically valuable [82,83]. In veterinary medicine, the habitual dependence on AI-generated explanations and diagnostic suggestions has been raised as a potential concern, as it may interfere with the development of independent clinical judgment. From a clinical perspective, excessive dependence on AI systems could reduce diagnostic vigilance, especially in complex or atypical cases where algorithmic performance is constrained by training data limitations. Ensuring that students develop the capacity to critically evaluate AI outputs is therefore essential not only for educational quality but also for patient safety and animal welfare [27].

Equity and access represent additional dimensions of concern. While AI technologies are often discussed as having the potential to democratize learning resources, unequal access to advanced AI tools during training may translate into disparities in clinical competences once graduates enter professional practice. Differences in AI literacy among veterinarians could further influence the consistency and reliability of AI-assisted decision making, potentially reinforcing inequalities in diagnostic quality and standards of care across institutions and regions. These risks underscore the importance of equitable training frameworks that ensure baseline competences in AI-supported clinical workflows for all students [84,85].

Algorithmic bias is consistently identified as a significant challenge in health-related AI applications, and its implications are particularly pronounced in veterinary medicine, where species diversity far exceeds that of human healthcare. Diagnostic systems trained on limited or non-representative datasets may perform suboptimally when applied to rare species, atypical morphologies or minority phenotypes. Professional guidelines therefore emphasize the need for the continuous auditing, validation and critical evaluation of AI systems used in veterinary contexts, especially those involved in diagnostic or decision support tasks [80]. Such biases may not only compromise diagnostic accuracy but also affect prevention strategies, epidemiological analyses and public health recommendations.

Data privacy, regulatory compliance and ethical data use further shape the responsible deployment of AI in veterinary education. AI tools frequently process sensitive information, including student data, clinical images and animal health records, placing veterinary institutions within broader legal and regulatory frameworks governing data protection. Professional bodies such as the Georgia Veterinary Medical Association stress full compliance with privacy laws and veterinary-specific regulations when AI is used in educational or clinical settings [86]. In Europe, the General Data Protection Regulation (GDPR) establishes requirements for data minimization, informed consent and algorithmic transparency that directly apply to AI-assisted educational platforms [87]. Additional guidance from the European Medicines Agency and the New Zealand Veterinary Association highlights the need to uphold rigorous ethical and operational standards when integrating AI into veterinary services [88,89]. Together, these frameworks emphasize the institutional responsibility to ensure compliance and to educate students about the ethical implications of data sharing and algorithmic processing.

Concerns related to academic integrity and professional accountability extend beyond the classroom and into clinical practice. If AI systems are used uncritically during training, similar patterns of dependence may persist after graduation, potentially affecting clinical responsibility and ethical standards. Maintaining clear boundaries between AI assistance and professional decision making is therefore regarded as important to ensure that veterinarians retain accountability for clinical judgments and uphold their ethical obligations toward animal health and welfare [54,80].

Student perceptions further highlight the need for transparent and ethically grounded AI integration. Surveys conducted across veterinary programs indicate that students express both enthusiasm for AI’s potential and concern regarding its limitations. Common expectations include formal instruction on AI use, clear institutional guidelines, transparency in AI-driven decision processes and safeguards against misuse [90,91]. Addressing these expectations is likely to require the embedding of AI literacy within veterinary curricula, fostering an open discussion of AI’s limitations and reinforcing critical interpretative skills. Taken together, the responsible integration of AI in veterinary education must extend beyond pedagogical innovation to encompass clinical safety, ethical accountability and animal welfare. By embedding ethical, regulatory and clinical considerations within structured educational frameworks, veterinary programs may help to ensure that AI adoption strengthens rather than undermines the quality and reliability of veterinary care. Core principles for responsible implementation include maintaining human-centered decision making, ensuring transparency in AI processes, mitigating algorithmic bias through representative datasets, promoting equitable access to AI tools and enabling the continuous evaluation of AI performance [90,91].

## 8. Conclusions and Future Directions

Artificial intelligence is rapidly redefining the landscape of veterinary education. Across diverse subject areas (from anatomy and parasitology to food science and veterinary public health), AI demonstrates considerable potential to support personalized learning, enhance diagnostic reasoning, automate assessments and integrate complex multimodal datasets. The breadth of examples reviewed in this article reveals that AI is no longer a peripheral technological supplement; it is increasingly being positioned as a relevant pedagogical instrument capable of enriching both theoretical understanding and practical skill development.

One of the most significant contributions of AI lies in its ability to personalize learning at scale. Adaptive platforms and intelligent tutoring systems analyze individual student needs, adjusting the level of difficulty, pacing and content presentation to optimize learning outcomes. This personalized approach is especially valuable in demanding subjects such as parasitology, anatomy and clinical reasoning, where stepwise scaffolding and differentiated instruction can substantially improve conceptual clarity, engagement and knowledge retention.

The multimodal capabilities of contemporary AI models represent a further pedagogical advantage. By integrating text, diagnostic images, ultrasound clips, laboratory data, audio recordings and video, AI systems mirror the cognitive processes required for real-world veterinary practice, where professionals must synthesize heterogeneous data sources to reach informed clinical decisions. These capabilities support immersive and authentic learning environments, particularly within anatomy, pathology and imaging-based training. Beyond individual clinical disciplines, AI also plays an increasingly important role in veterinary public health, epidemiology and food safety education. Predictive models, simulation-based exercises and AI-supported surveillance tools enhance students’ understanding of disease transmission, outbreak dynamics and risk assessment. In food science and hygiene, AI-driven virtual laboratories and predictive microbiology platforms allow learners to explore microbial behavior, process optimization and safety management strategies without exposure to real-world hazards, reinforcing both technical competence and safety awareness.

Despite these pedagogical benefits, the successful integration of AI into veterinary curricula requires a deliberate, evidence-based and ethically grounded approach. Concerns related to algorithmic bias, overreliance on automated systems, data privacy, equity of access and academic integrity must be systematically addressed to ensure that AI enhances rather than compromises educational quality and clinical preparedness. Importantly, AI tools must support—not replace—professional judgment. Human oversight, the critical evaluation of AI-generated outputs and explicit instruction in AI literacy are essential components of a responsible integration strategy.

The primary contribution of this review lies in synthesizing current developments in generative and multimodal AI, contextualizing them within the specific requirements of veterinary education and highlighting the challenges that must be addressed to enable responsible adoption. Despite the growing body of literature on artificial intelligence in education and health sciences, an important limitation identified in the current field is the scarcity of robust quantitative data specifically addressing the adoption and use of AI technologies in veterinary and animal sciences. Most available quantitative evidence is derived from broader studies in higher education or biomedical disciplines, within which veterinary education is often underrepresented or aggregated. This gap highlights the need for dedicated, discipline-specific quantitative research to accurately assess AI uptake, usage patterns and educational or clinical impact in veterinary contexts.

Looking ahead, several interconnected priorities emerge. There is a clear need for empirical research evaluating the educational effectiveness of AI tools in authentic veterinary teaching contexts, including their impacts on learning outcomes, diagnostic accuracy, skill acquisition and long-term retention. At the same time, AI literacy and critical digital competences should be formally embedded within veterinary curricula, empowering students to understand AI mechanisms, recognize its limitations and critically appraise algorithmic recommendations. Future innovations should also prioritize the development of transparent, ethically aligned and domain-specific AI models tailored to the diversity of veterinary practice. Given the wide range of species, breeds and clinical scenarios encountered in veterinary medicine, AI systems must be trained on representative datasets to mitigate biases and enhance diagnostic reliability. Open and customizable AI frameworks offer promising pathways for achieving these goals. In parallel, continued collaboration between veterinary institutions, regulatory bodies and professional organizations will be essential to establish clear guidelines governing AI use in education and clinical training, ensuring compliance with data protection regulations and professional standards.

Ultimately, as AI becomes increasingly embedded within veterinary education and practice, maintaining a human-centered approach remains paramount. Veterinary training must reinforce the central role of human expertise in clinical reasoning, ethical decision making and animal welfare. Framing AI as an assistive technology that augments, rather than replaces, professional judgment will be critical in safeguarding clinical responsibility and sustaining trust in veterinary care.

## Figures and Tables

**Table 1:** Technological capabilities and pedagogical utility of major AI systems for veterinary teaching and research.

Model/Provider	Key Strengths	Educational Applications (Veterinary Focus)	Unique Advantages
Anthropic (Claude 4)	Emphasis on safety, factual accuracy and transparent reasoning; very large context window (≈200 k tokens)	Long-form tutoring; analysis of textbooks, case files and multi-section documents; support for higher-order thinking	Reduced hallucination rates; excellent for sustained technical dialog [38,39]
DeepSeek (V3, R1)	High technical efficiency at significantly lower cost	Computational modeling; data processing in epidemiology and food science; reasoning support	DeepSeek V3 strong in data analysis; DeepSeek R1 excels in logic tasks; ideal for resource-limited programs [41]
Google (Gemini 2.5)	Highly sophisticated multimodal processing; strong technical document handling	Analysis of medical/scientific imagery; extraction and summarization of academic texts; support for structured learning materials	Deep integration with Google Workspace facilitates institutional deployment [37]
Meta (Llama 4)	Extraordinary context window (up to 10 M tokens); open-source adaptability	Processing of entire textbooks or long clinical transcripts; customizable models for species-specific or curricular needs	Campus-controlled customization and vocabulary tuning; strong for privacy-sensitive environments [42]
Microsoft (Copilot and Copilot Chat)	Embedded within Office 365 ecosystem; productivity-oriented	Summarization of documents; creation of teaching materials; analysis of assignments; support for course planning and admin tasks	Seamless availability for institutions already using Word, PowerPoint, Teams, OneNote [40]
OpenAI (GPT-4, GPT-4o)	Native multimodality (text, images, audio); strong domain reasoning	Interpretation of radiographs; analysis of lab results; explanation of clinical images; support in anatomy and case-based learning	GPT-4o enables real-time multimodal processing for integrated clinical scenarios and anatomical visualizations [7]

## Data Availability

No new data were created or analyzed in this study.
